# Aerobic exercise capacity is maintained over a 5-year period in mild-to-moderate chronic kidney disease: a longitudinal study

**DOI:** 10.1186/s12882-020-02110-2

**Published:** 2020-11-11

**Authors:** Helena Wallin, Eva Jansson, Carin Wallquist, Britta Hylander Rössner, Stefan H. Jacobson, Anette Rickenlund, Maria J. Eriksson

**Affiliations:** 1 Department of Laboratory Medicine, Division of Clinical Physiology, Karolinska Institutet, Stockholm, Sweden; 2 Department of Clinical Physiology, Karolinska University Hospital, Stockholm, Sweden; 3 Department of Molecular Medicine and Surgery, Karolinska Institutet, Stockholm, Sweden; 4 Department of Nephrology, Karolinska University Hospital, Karolinska Institutet, Stockholm, Sweden; 5 Division of Nephrology, Department of Clinical Sciences, Karolinska Institutet Danderyd University Hospital, Stockholm, Sweden

**Keywords:** Exercise capacity, Non-dialysis chronic kidney disease, Peak heart rate, Physical activity

## Abstract

**Background:**

Aerobic exercise capacity is reduced in non-dialysis chronic kidney disease (CKD), but the magnitude of changes in exercise capacity over time is less known. Our main hypothesis was that aerobic ExCap would decline over 5 years in individuals with mild-to-moderate CKD along with a decline in renal function. A secondary hypothesis was that such a decline in ExCap would be associated with a decline in muscle strength, cardiovascular function and physical activity.

**Methods:**

We performed a 5-year-prospective study on individuals with mild-to-moderate CKD, who were closely monitored at a nephrology clinic. Fiftytwo individuals with CKD stage 2–3 and 54 age- and sex-matched healthy controls were included. Peak workload was assessed through a maximal cycle exercise test. Muscle strength and lean body mass, cardiac function, vascular stiffness, self-reported physical activity level, renal function and haemoglobin level were evaluated. Tests were repeated after 5 years. Statistical analysis of longitudinal data was performed using linear mixed models.

**Results:**

Exercise capacity did not change significantly over time in either the CKD group or controls, although the absolute workloads were significantly lower in the CKD group. Only in a CKD subgroup reporting low physical activity at baseline, exercise capacity declined. Renal function decreased in both groups, with a larger decline in CKD (*p* = 0.05 between groups). Peak heart rate, haemoglobin level, handgrip strength, lean body mass and cardiovascular function did not decrease significantly over time in CKD individuals.

**Conclusions:**

On a group level, aerobic exercise capacity and peak heart rate were maintained over 5 years in patients with well-controlled mild-to-moderate CKD, despite a slight reduction in glomerular filtration rate. In line with the maintained exercise capacity, cardiovascular and muscular function were also preserved. In individuals with mild-to-moderate CKD, physical activity level at baseline seems to have a predictive value for exercise capacity at follow-up.

## Background

We and others have previously shown that aerobic exercise capacity (ExCap), measured as peak oxygen uptake (VO_2_peak) or peak ExCap, is already reduced in the early stages of non-dialysis chronic kidney disease (CKD) [1–3]. Furthermore, reduced ExCap is associated with increased mortality and morbidity in CKD [4, 5]. Aerobic ExCap is influenced by the function of most organ systems, particularly the cardiovascular and muscular systems. The cause of the reduced ExCap in CKD is likely multifactorial and may differ between different stages of CKD. Anaemia, autonomic dysfunction, vascular dysfunction, cardiac and skeletal muscle abnormalities may all contribute to exercise intolerance in non-dialysis CKD [1, 3, 6–10]. We recently showed that aerobic ExCap in patients with mild to severe CKD stages 2–5 was strongly associated with peak heart rate (HR), haemoglobin level and stroke volume [1].

Although renal function declines over time in most individuals with CKD, the rate of progression differs, and some individuals do not show any progression at all [11, 12]. Surprisingly little is known about how aerobic ExCap and muscle strength and mass change over time in individuals with non-dialysis CKD and how this correlate with changes in renal function. Leikis et al. found that in patients with CKD stages 3–4, both leg strength and VO_2_peak declined over a two-year period as glomerular filtration rate (GFR) declined, despite stable haemoglobin levels [13]. The progression of muscle wasting in CKD seems to be highly variable, although few data are available [14]. Physical activity levels of individuals with CKD are generally low and are increasingly reduced in the later stages of CKD [15, 16]. A sedentary lifestyle most likely contributes to a further reduction in aerobic ExCap in patients with CKD, and vice versa.

Given the high incidence of cardiovascular disease in CKD [17] and the higher mortality associated with reduced aerobic ExCap [4, 18], it is of interest to monitor aerobic ExCap. Our main hypothesis was that aerobic ExCap would decline over 5 years in individuals with mild-to-moderate CKD along with a decline in renal function. A secondary hypothesis was that such a decline in ExCap would be associated with a decline in muscle strength, cardiovascular function and physical activity. To address this, we investigated individuals with mild-to-moderate CKD, who were closely monitored at a nephrology clinic.

## Methods

### Study protocol

The current study (PROGRESS 2002) is part of a prospective observational cohort study that has been described by our group in more detail previously [8]. Fifty-two patients with non-dialysis, mild-to moderate CKD, stage 2–3 (aged 18–65 years) and 54 healthy controls, age- and sex-matched with the CKD group, were included. All subjects had participated in exercise testing at baseline. The cohort was monitored during five years. Recruitment of patients was done consecutively from the Department of Renal Medicine at the Karolinska University Hospital during 2002–2009. Patients were recruited if they fulfilled the criteria for CKD stage 2–3a as defined by the National Kidney Foundation [19]. The controls were recruited during the same time-period using two different approaches; they were either randomly selected from the Swedish Total Population Register (*n* = 31) or recruited through the website of the Karolinska University Hospital (*n* = 23). As previously stated [1, 8], the exclusion criteria for CKD patients and controls were: active infection, current immunosuppressive therapy with steroids or cytotoxic drugs, current malignancy, kidney transplantation or kidney donation, or blood-transmitted disease. Additional exclusion criteria for the controls were history of kidney disease, diabetes, cardiovascular disease or current medication.

GFR was determined by iohexol plasma clearance [20]. During follow-up, GFR measurement by iohexol was repeated in the CKD group. In both CKD and controls, the Chronic Kidney Disease–Epidemiology Collaboration (CKD–EPI) equation was used to assess estimated GFR (eGFR) [21]. Methodological considerations regarding eGFR have been previously reported [22].

All included subjects underwent testing at baseline and after three and five years at the Department of Nephrology and the Department of Clinical Physiology at Karolinska University Hospital. The CKD group was regularly followed at the Department of Renal Medicine where the goal was to aggressively treat hypertension, hyperlipidaemia and proteinuria. The results of the cardiovascular function tests from these three test occasions have been reported previously [23]. However, since there were few controls who performed an exercise test at the 3-year follow-up, we have chosen to only report the results from baseline and the 5-year-follow-up in this paper. The controls were more actively encouraged to participate in all follow-up-tests at this later follow-up, which resulted in comparable numbers of subjects in both groups.

**Fig. 1:**
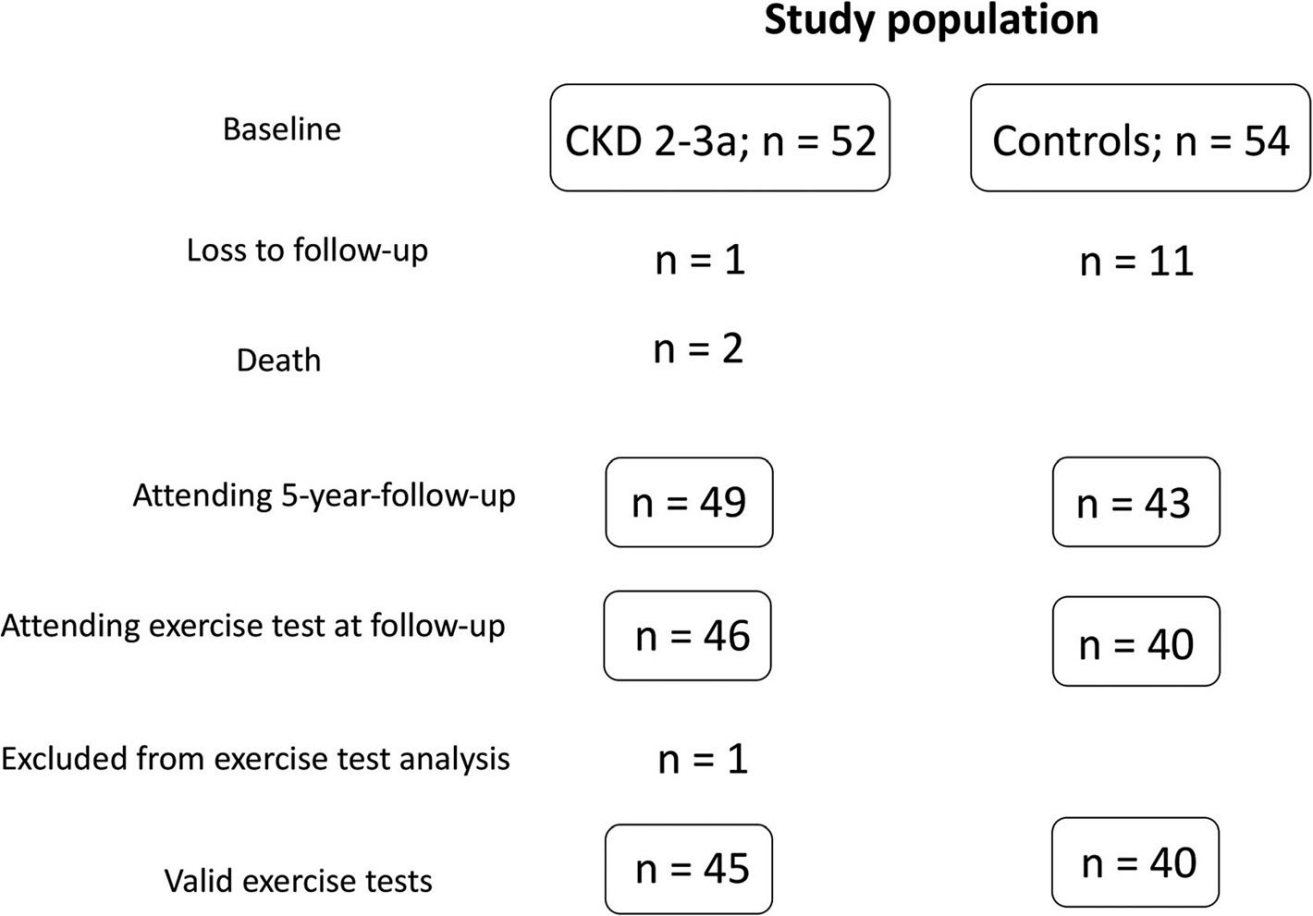
Study population at baseline and 5-year-follow up

The study population and the excluded individuals are presented in Fig. 1. Two participants in the CKD group died from cancer before the end of the follow-up period. No patient in the CKD group required renal replacement therapy during the follow-up period. A few patients were lost to follow-up. Forty-nine participants in the CKD group and 43 in the control group participated in the 5-year follow-up. Forty-six participants in the CKD group and 40 in the control group performed an exercise test; but one participant with CKD was excluded from the follow-up exercise test analysis because the exercise test was terminated early owing to the occurrence of atrial fibrillation with a high HR. Two other participants with CKD had atrial fibrillation before and during the exercise test; therefore, these individuals were not included in the peak HR analyses.

The study protocol was reviewed and approved by the Local Ethics Committee and Institutional Review Board of the Karolinska Institutet at the Karolinska University Hospital. All participants gave their written informed consent.

### Measurements

#### Aerobic exercise capacity

Exercise testing was performed as an incremental test on a cycle ergometer (RE990; Rodby Innovation AB, Uppsala, Sweden) with a protocol consistent with current standards at our institution, as described in detail in our previous study [1]. Participants exercised until exhaustion and the highest perceived exertion was rated according to the Borg CR10 scale [24]. The peak workload in watt (W) was used as a measure of aerobic ExCap.

#### Muscular function

Handgrip strength was measured as maximum voluntary isometric contraction with a handheld dynamometer (Grip-A; Takei Scientific Instruments Co., Ltd., Tokyo, Japan) with the subject standing. The test was carried out three times in the dominant upper extremity, with the highest value being reported as handgrip strength.

#### Cardiac and vascular function

Transthoracic echocardiography was performed according to present recommendations [25]. The ultrasound equipment (Sequoia 512; Siemens Medical Solutions, Mountain View, CA, USA) was used for evaluation of cardiac function which was expressed accordingly: systolic left-ventricular (LV) function as ejection fraction (EF), right-ventricular systolic function as tricuspid annular plane systolic excursion (TAPSE) and diastolic LV function as the E/é ratio (mitral flow velocity E, divided by tissue LV velocity é). Arterial stiffness was assessed through ultrasound of the carotid arteries. The pressure strain elastic modulus (Ep) was estimated as previously described [8].

#### Body composition

A whole-body dual-energy X-ray absorptiometry scan was performed to measure body composition (QDR 4500 Discovery A, software version 12.3; Hologic, Bedford, MA, USA). Only the CKD group were examined at follow-up.

#### Physical activity level

Study subjects rated their physical activity level using a four-point scale modified from the Saltin–Grimby Physical Activity Level Scale [26]. As previously described by us, the modification was as follows [1]:

‘Level 1 = *Regular exercise*: running, swimming, tennis, badminton, gymnastics or similar activity on three or more occasions per week; every session should last at least 30 min and cause sweating.

Level 2 = *Moderate amount of regular exercise*: running, swimming, tennis, badminton, gymnastics or similar activity on 1–2 occasions per week; every session should last at least 30 min and cause sweating.

Level 3 = *Light exercise*: walking or cycling or other physical activity during at least 2 h per week, usually without sweating; this includes walking or cycling to/from work, Sunday walks, gardening, fishing, table tennis and bowling or similar activity.

Level 4 = *Sedentary*: mostly reading, watching television, movies or other sedentary activities, or walking, cycling or light exercise for less than 2 h per week.’(p.5–6).

In one of the analyses, due to the small sample size, the four physical activity levels at baseline were merged into two new subgroups; high (physical activity level 1 and 2) and low (physical activity level 3 and 4) physical activity.

#### Blood samples

Venous blood samples were obtained at the Karolinska University Laboratory for measurement of haemoglobin, creatinine and high-sensitivity C-reactive protein (hs-CRP) concentrations. Blood samples were taken at the inclusion visit.

### Statistical analysis

Data is presented as number, percentage, mean and standard deviation, or median and interquartile range for skewed variables. The normality assumption was assessed graphically using histograms. Potential outliers were examined graphically by box plots and their validity were assessed. Logarithmic transformation was performed for variables with a skewed distribution when they were analysed in mixed models. Student’s *t*-test, Mann–Whitney *U*-test or chi-square test, where applicable, were used to compare the groups at baseline. A *p*-value < 0.05 for a two-tailed test was considered statistically significant.

Linear mixed models were considered to be the most suitable method for the analysis of longitudinal data because this method provides effect estimates from the whole study sample provided that the values missing at follow-up are random. Changes over time were analysed separately in the CKD group and in the controls and the changes were compared between the groups by the interaction factor group × time in the mixed model. The analyses were adjusted for different background factors (covariates), including age at baseline, sex, height and beta-blocker use. The choice of covariates depended on the chosen outcome variable. Changes over time were also analysed in physical activity subgroups.

A two-way repeated measures ANOVA was performed for the main outcome variable aerobic ExCap as part of a sensitivity analysis.

To analyse longitudinal data where the outcome variables were ordered categoricals, generalized estimating equations (GEE) model were used. The model was set up with the same factors as in the mixed linear model mentioned above. The parameter estimates from the GEE model are presented as odds ratios (OR) and 95% confidence intervals (CI).

Pearson (r) or Spearman (r_s_) correlation coefficients were used to analyse the relationship between two variables.

Statistical analyses were performed using IBM SPSS Statistics (version 23.0; IBM, Armonk, NY, USA) and SAS (version 9.3; SAS Institute, Cary, NC, USA) software.

## Results

Table 1 shows the baseline characteristics of the study subjects. The mean age of the whole cohort at entry was 47 years and there were no significant differences between the CKD group and controls in age, sex or body size.

**Table 1:** Baseline characteristics

Variable	Controls	CKD	***p***-value
Subjects (n)	54	52	
Age (years)	48 ± 11	47 ± 11	0.7
Male, n (%)	33 (61)	32 (61)	0.8
Height (cm)	176 ± 9	174 ± 9	0.4
Weight (kg)	77 ± 12	76 ± 16	1
BMI (kg/m^2^)	24.9 ± 3.4	25.1 ± 4.0	0.2
Lean body mass (kg)	54 ± 11	52 ± 11	0.5
GFR (mL/min/1.73 m^2^)	99 ± 12	60.3 ± 5.2	< 0.001
eGFR (mL/min/1.73 m^2^)	96 ± 13	59 ± 13	< 0.001
Haemoglobin (g/dL)	14.2 ± 1.2	13.5 ± 1.4	0.02
Hs-CRP (mg/L)	0.89 (0.47–2.1)	1.60 (0.86–3.5)	0.04
Diabetes, n (%)		10 (22)	NA
24-h SBP (mmHg)	124 ± 11	122 ± 14	0.4
24-h DBP (mmHg)	78 ± 8	76 ± 7	0.4
***Aetiology of CKD, n (%)***			
Familial/hereditary/congenital disease		14 (27)	NA
Primary glomerulonephritis		17 (33)	NA
Secondary glomerular/systemic disease		9 (17)	NA
Miscellaneous/unknown		12 (23)	NA
***Medication, n (%)***			
Beta blocker		10 (19)	NA
Diuretics		12 (23)	NA
ACE inhibitors		22 (42)	NA
Angiotensin II blockers		21 (39)	NA
Calcium-channel blockers		10 (19)	NA
ESA		1 (2)	NA
Oral cortisone		2 (4)	NA
Iron supplementation		4 (8)	NA
***Functional measurements***			
Peak workload (W)	238 ± 60	193 ± 63	< 0.001
Peak HR (bpm)	177 ± 11	161 ± 24	< 0.001
Peak RPE	8.6 ± 1.3	9 ± 1.3	0.2
Handgrip strength (kg)	44 ± 12	40 ± 11	0.08

*BMI* body mass index; *bpm* beats per minute; *CKD* chronic kidney disease; *eGFR* glomerular filtration rate estimated by CKD–EPI; *ESA* erythropoiesis-stimulating agents; *GFR* glomerular filtration rate measured by iohexol clearance; *HR* heart rate; *hs-CRP* high-sensitivity C-reactive protein; *n* number; *24-h SBP*/*DBP* average 24-h systolic/diastolic blood pressure

Values reported as number (percentage), mean ± standard deviation or median (interquartile range). *P*-value: *t*-test, Mann–Whitney *U*-test (continuous variables) or chi-square (categorical values)

Based on their GFR, 31 individuals in the CKD group were in CKD stage 2 and 21 in CKD stage 3 at baseline. At follow-up, two individuals in the CKD group were in stage 1; nine in stage 2; 27 in stage 3; five in stage 4; and one was in stage 5. Ten individuals in the CKD group had diabetes at inclusion and follow-up and two in the control group had developed diabetes at follow-up. At baseline, 10 individuals in the CKD group were on beta-blocker treatment. At follow-up, this number had increased to 15 and four of the controls had started beta-blocker treatment.

### Renal function and blood analyses

At baseline, GFR measured by iohexol clearance was 60.3 ± 5.2 mL/min/1.73 m^2^ in CKD and 99.5 ± 12.5 mL/min/1.73 m^2^ in the controls (*p* <  0.001). At follow-up, measured GFR in the CKD group was 50.2 ± 16.9 mL/min/1.73 m^2^, a mean decline of 17% over 5 years. eGFR significantly decreased during the follow-up period in both groups; The difference in the change in eGFR over time between the groups was borderline significant (interaction group × time, *p* = 0.05) (Table 2). The annual rate of decline in eGFR was 1.8 mL/min/1.73 m^2^ in the CKD group and 0.8 mL/min/1.73 m^2^ in the control group.

**Table 2:** Functional measurements at baseline and 5-year follow-up

	*Controls*			*CKD*					
	Baseline (*n* = 54)	Year 5	***P***-value time^**a**^	Baseline (*n* = 52)	Year 5	***P***-value time^**a**^	***P***-value group^**b**^	***P***-value group*time^**c**^	Covariates^**d**^
Peak workload (W)	237 (225–249)	233 (221–245)	0.3	195 (184–207)	190 (178–202)	0.2	< 0.001/< 0.001	0.8	Age, sex, height
		*n* = 40			*n* = 45				
Peak heart rate (bpm)	175 (171–179)	171 (167–176)	0.04	161 (157–165)	160 (155–164)	0.3	< 0.001/0.001	0.4	Age, BB medication
		*n* = 40			*n* = 43				
HR reserve (bpm)	99 (94–104)	102 (96–107)	0.4	91 (86–96)	89 (84–95)	0.5	0.03/0.002	0.2	Age, BB medication
		*n* = 40			*n* = 43				
Handgrip strength (kg)	44(43–46)	43 (41–44)	0.006	41 (39–42)	41 (39–42)	0.8	< 0.001/0.07	0.06	Age, sex, height
		*n* = 40			*n* = 48				
LV systolic function LVEF (%)	65 (63–68)	61 (59–64)	< 0.001	62 (60–65)	61 (58–63)	0.1	0.06/0.7	0.1	*
		*n* = 41			*n* = 45				
LV diastolic function E/é	5.0 (4.7–5.4)	5.7 (5.3–6.1)	< 0.001	5.6 (5.3–6.0)	5.9 (5.6–6.3)	0.06	0.01/0.3	0.1	Age
		*n* = 43			*n* = 46				
RV systolic function TAPSE (cm)	2.5 (2.4–2.6)	2.5 (2.4–2.6)	0.4	2.3 (2.2–2.4)	2.4 (2.3–2.5)	0.09	0.06/0.2	0.6	*
		*n* = 43			*n* = 45				
Vascular function Ep ((N/m^2^) × 10^4^)^e^	5.8 (5.4–6.3)	6.0 (5.5–6.6)	0.5	6.5 (5.8–7.1)	6.8 (6.2–7.4)	0.4	0.06/0.08	1	Age
		*n* = 43			*n* = 47				
eGFR (mL/min/1.73 m^2^)	96 (92–100)	92 (87–96)	0.03	59 (55–62)	50 (46–54)	< 0.001	< 0.001/< 0.001	0.05	
		*n* = 43			*n* = 47				
Haemoglobin (g/dL)	14.2 (13.8–14.5)	14.7 (14.3–15.0)	0.002	13.6 (13.3–13.9)	13.9 (13.6–14.2)	0.04	0.01/0.001	1	Sex
		*n* = 40			*n* = 49				
Hs-CRP (mg/L)^e^	1.0 (0.7–1.3)	1.0 (0.8–1.3)	0.9	1.9 (1.4–2.6)	1.8 (1.4–2.4)	0.8	0.002/0.006	0.8	
		*n* = 43			*n* = 49				

*BB* beta-blocker; *bpm* beats per minute; *CKD* chronic kidney disease; *E/é* left-ventricular early filling velocity/early diastolic myocardial velocity (a variable of left-ventricular diastolic function); *eGFR* glomerular filtration rate estimated by CKD–EPI; *Ep* pressure strain elastic modulus in the carotid artery; *HR* heart rate; *hs-CRP* high-sensitivity C-reactive protein; *LV* left ventricular; *LVEF* left-ventricular ejection fraction; *n* number; *RV* right ventricular; *TAPSE* tricuspid annular plane systolic excursion (a variable of right-ventricular systolic function)

Values reported as mean (95% confidence interval). *P*-value: linear mixed models. Mean values are adjusted for covariates

^a^Significance of the change over time in the control group and the CKD group, respectively

^b^Significance of the difference between the control group and the CKD group at baseline/year 5

^c^Significance of the difference in change over time between the CKD group and the control group

^d^Baseline values are used as fixed covariates

^e^Values presented in the table are the calculated anti-logs of the log-scale estimates that were used in linear mixed models analyses

* Age was tested as a covariate but was not significant

Haemoglobin was significantly lower in the CKD group than in controls at baseline and follow-up. There was a small but significant increase over time in both groups. This change did not differ significantly over time between the groups (Table 2). However, separate analyses in men and women using the paired samples *t*-test showed that haemoglobin increased significantly in women (*p* = 0.001) but not in men (*p* = 0.2).

Hs-CRP was higher in CKD than in controls at both baseline and follow-up, but did not significantly change over time in CKD or controls.

### Exercise test results

Aerobic ExCap and peak HR were both significantly lower in CKD than in controls at both time points, and did not change significantly over time in neither CKD nor controls (Table 2, mixed model with values adjusted for covariates). The effect size (repeated measures) for the differences between the groups in ExCap was 0.16, calculated according to Lenhard and Lenhard ([27]). In CKD, there was a non-significant average decline in ExCap of 2.6%. The non-significant decline in peak HR in the CKD group was 0.6%. In controls, the mean values of ExCap showed a non-significant decrease of 1.7% and peak HR significantly decreased by an average of 2.3%. Inclusion of covariates in the analyses did not significantly change the results. There was no significant difference in the rating of perceived exertion (RPE) between the groups at baseline. At follow-up, the mean ratings were 8.3 for CKD and 8.5 for controls (*p* = 0.7 between groups).

The change in ExCap over time was also analysed using a two-way ANOVA, showing no significant difference between the CKD (*n* = 45) and control group (*n* = 40), *p*-value for interaction time*group was 0.831.

### Muscle strength and lean body mass

Handgrip strength was significantly lower in CKD than in controls at baseline, but not at follow-up (Table 2, mixed model). The difference in the change over time between the two groups was borderline significant (*p* = 0.06), with a significant reduction in the control group. There was no significant difference in LBM between groups at baseline (CKD 52.8 kg; controls 53.5 kg, *p* = 0.3, analysed by linear mixed model) and LBM increased from 52.8 kg to 54.5 kg (*p* = 0.02) at follow-up in the CKD group. Mean values were adjusted for the covariates of age at baseline, sex, height and weight at baseline. Weight increased from 75.7 kg to 78.3 kg (*p* = 0.006, paired samples t-test) in the CKD group.

### Cardiovascular function

At baseline, LV diastolic function (E/é ratio), was significantly lower in the CKD group compared with controls, although within normal values; while LV systolic function (EF), right ventricular systolic function (TAPSE) and vascular stiffness (Ep) did not differ significantly between groups (Table 2, mixed model). E/é, EF, TAPSE and Ep did not change significantly over 5 years in the CKD group, nor was the change over time significantly different from that in controls (Table 2). E/é increased and EF decreased in controls from baseline to follow-up with values remaining within the normal range.

### Self-reported physical activity

Self-reported physical activity levels differed between groups at baseline with controls being physically more active than CKD patients (Tables 3, 4, 5). At follow-up, there was no significant difference in physical activity level between groups. Individuals with CKD reported higher physical activity levels at follow-up compared with baseline while controls reported lower physical activity levels at follow-up compared with baseline. The interaction group x time was statistically significant (*p* = 0.0002), indicating that the two groups differed regarding their change in physical activity level over time.

**Table 3:** Physical activity (PA) level at baseline and 5-year follow-up

	*Baseline*		*Year 5*	
	CKD, ***n*** = 48	Controls, *n* = 40	CKD, ***n*** = 48	Controls, ***n*** = 40
PA level 1	10	14	16	9
PA level 2	15	19	14	12
PA level 3	19	15	15	16
PA level 4	8	0	3	3

*CKD* chronic kidney disease; *n* number

Only individuals who reported physical activity levels at both time-points are included

**Table 4:** Change in physical activity (PA) level between baseline and 5-year follow-up

	CKD, ***n*** = 48	Controls, ***n*** = 40
Increased PA level	16	4
Decreased PA level	5	15
Unchanged PA level	27	21

*CKD* chronic kidney disease; *n* number

Only individuals who reported physical activity levels at both time-points are included

**Table 5:** Odds ratios for change in physical activity (PA) level between baseline and 5-year follow-up

	Odds ratio	Confidence interval	***P***-value
^a^CKD: Follow-up vs. baseline	2.12	1.26–3.56	0.005
^b^Controls: Follow-up vs. baseline	0.54	0.35–0.84	0.006
^c^CKD vs. controls at baseline	0.42	0.21–0.86	0.02
^d^CKD vs. controls at follow-up	1.66	0.77–3.63	0.2

*CKD* chronic kidney disease

^a^CKD: Odds for increased PA at follow-up compared with baseline

^b^Controls: Odds for increased PA at follow-up compared with baseline

^c^Odds for higher PA in CKD group compared with controls at baseline

^d^Odds for higher PA in CKD group compared with controls at follow-up. Results are analysed using a generalized estimation equation. The results are based on 188 observations from 106 subjects. *P*-value: chi-square test

There were significant correlations between physical activity level and ExCap for both the CKD group (r_s_ = − 0.4, *p* = 0.001, *n* = 52 at baseline and r_s_ = − 0.4, *p* = 0.01, *n* = 45 at follow-up) and controls (r_s_ = − 0.4, *p* = 0.01, *n* = 48 at baseline and r_s_ = − 0.4, p = 0.01, *n* = 40 at follow-up) where being more physically active correlated to higher ExCap.

We stratified the subjects into high and low physical activity at baseline as described in Methods. The two CKD subgroups had similar GFR; 60.4 (SD 5.5) and 60.3 (SD 5.0) mL/min/1.73 m^2^ for high and low physical activity, respectively. In the CKD patients, individuals with low physical activity at baseline significantly decreased in ExCap, while individuals with high physical activity did not (Table 6). In the control group, there were no significant differences in the change in ExCap between individuals with high and low physical activity at baseline.

**Table 6:** Aerobic exercise capacity at baseline and 5-year follow-up based in physical activity subgroups

	*High PA level baseline*			*Low PA level baseline*					
	Baseline	Year 5	***P***-value time^**a**^	Baseline (*n* = 48)	Year 5	***P***-value time^**a**^	***P***-value group^**b**^	***P***-value group*time^**c**^	Covariates^**d**^
**CKD**									
Peak workload (W)	217 (200–234) *n* = 25	218 (201–235)	0.8	172 (155–188) *n* = 27	160 (143–176)	0.005	< 0.001/< 0.001	0.03	Age, sex, height
**Controls**									
Peak workload (W)	250 (237–262) *n* = 33	247 (233–261)	0.7	227 (207–246) *n* = 13	221 (201–241)	0.5	0.06/0.04	0.7	Age, sex, height

*PA* physical activity: *CKD* chronic kidney disease

^a^Significance of the change over time in the group with high PA level and the group with low PA level, respectively

^b^Significance of the difference between the group with high PA level and the group with low PA level at baseline/year 5

^c^Significance of the difference in change over time between the group with high PA level and the group with low PA level

^d^Baseline values are used as fixed covariates

### Correlations between the change in ExCap and physiological measurements

Correlations between the change in aerobic ExCap and changes in selected physiological and biochemical measurements were analysed for the CKD group. There were significant correlations between the change in ExCap and changes in peak HR and handgrip strength (r = 0.5 and 0.3 respectively, *p* = 0.001 and 0.03 respectively, *n* = 43 and 45 respectively). The change in measured GFR was significantly correlated with the change in aerobic ExCap in CKD (r = 0.3, *p* = 0.03, *n* = 42). However, when eGFR was used, the correlation between the change in eGFR and the change in ExCap was not significant (r = 0.06 and *p* = 0.7, *n* = 44). The change in physical activity level did not correlate to the change in ExCap (r_s_ = − 0.05 and *p* = 0.7, *n* = 45), nor did the change in haemoglobin level, Hs-CRP level, E/é or EP (r = 0.1, 0.03, − 0.08 and − 0.01 respectively, *p* = 0.4; 0.9, 0.6 and 0.9, respectively, n = 45, 42, 43 and 43, respectively). For controls, there were no significant correlations between the change in ExCap and changes in peak HR, handgrip strength, physical activity level, and eGFR (r = 0.1, 0.1, − 0.2 and − 0.3 respectively, *p* = 0.5, 0.5, 0.2 and 0.06, respectively, *n* = 40, 40, 40 and 39, respectively).

## Discussion

Changes over a 5-year period in aerobic ExCap and known determinants were analysed in individuals with mild-to-moderate CKD and compared with healthy individuals. Individuals with CKD were attended a nephrology clinic and were closely monitored. In these patients, aerobic ExCap, peak HR, handgrip strength, LBM, haemoglobin level and cardiovascular function were all maintained over 5 years, despite a 17% reduction in GFR over time. However, in a subgroup of CKD patients who reported low physical activity level at baseline, ExCap significantly decreased over 5 years.

We believe that this is the first study to evaluate the change in aerobic ExCap over time in CKD patients compared with healthy controls. As expected, eGFR decreased more in CKD patients than in controls (*p* = 0.05). Aerobic ExCap is expected to decline with age, with an average 5–20% decline per decade in cross-sectional studies of healthy individuals, while longitudinal studies show a greater decline in older individuals compared with younger people [28, 29]. The mean change in ExCap in our study was almost 3% over 5 years in the CKD group and a little less in the control group. Our study included some younger individuals (< 35 years old), which could explain the modest decline compared with those seen in previous studies of healthy individuals. An important finding from our study is that ExCap did not decline more in the CKD group attending a nephrology clinic than in the controls. Reductions in peak HR and peripheral oxygen utilization but not stroke volume appear to mediate the normal age-associated decline in aerobic ExCap [28–30]. The expected decline in peak HR with normal aging is not as pronounced as the decline in aerobic ExCap of 4–6% per decade that was reported by Fleg et al. [30], which is similar to our data for the controls. The reduction in peak HR in the CKD group was only 0.6%. For the CKD group, there was a significant correlation between the change in ExCap over 5 years and the change in peak HR, which was not seen in the control group. This suggests that peak HR, or the possibility of achieving a high peak HR, could be an important factor influencing ExCap in this CKD group. Peripheral oxygen utilization and maximal stroke volume were not measured in our study, however a measure of diastolic function, E/é, decreased in controls but not in CKD patients over 5 years. Diastolic dysfunction may lead to an inability to increase stroke volume at exercise through the Frank–Starling mechanism and a preserved diastolic function at rest might indicate that maximal stroke volume was also preserved.

While both aerobic ExCap and peak HR are known to be more reduced in the later stages of CKD than in the earlier stages [1, 3, 31], only one small study has reported the changes in aerobic ExCap over time in non-dialysis CKD patients [13]. That study found a reduction in VO_2_peak of 9% over two years, a stable haemoglobin level and a fall of 28% in the calculated creatinine clearance. As a decline in leg strength paralleled the reduction in VO_2_peak, the authors speculated that the reduction in VO_2_peak was likely related to intrinsic muscle changes. However, peak HR decreased with a mean value of 9 beats per minute over only two years, which may also have influenced aerobic ExCap in that study. The difference between the results of our study compared with that study may in part be explained by the higher mean GFR in our study (at baseline, 60 versus 31 mL/min/1.73 m^2^) and the smaller decline in GFR over time. Furthermore, handgrip strength and LBM did not decrease over 5 years in the CKD group in our study. Grip strength is known to be reduced in non-dialysis CKD patients [1, 32, 33] and to further decrease in conjunction with the progression of CKD [33]. Muscle wasting seems to be more pronounced in patients with dialysis-dependent CKD than in those with non-dialysis CKD [34]. However, John et al. [14] showed a highly variable course of the change in muscle cross-sectional area over two years, and the rate of muscle loss was actually more pronounced in non-dialysis CKD patients than in patients on dialysis. Compared with the non-dialysis CKD group in our study, the non-dialysis CKD group in the study by John et al. had more advanced CKD with a substantially lower eGFR (16 mL/min/1.73 m^2^), which might explain the discrepancy between the results.

The degree of physical activity is an important factor associated with exercise capacity, which we previously showed for CKD stages 2–5 [1] and physical activity was indeed correlated to ExCap in both the CKD group and in the controls in our study. There was no significant correlation between the change in physical activity level and the change in ExCap in CKD. Nonetheless, the maintained or increased physical activity level may have contributed to the maintenance of their ExCap.

Our finding of a decline in ExCap in the CKD patients reporting low physical activity at baseline is important considering the relevance of physical function for quality of life and the increased mortality associated with reduced ExCap [4]. It is also noteworthy that the CKD group with low physical activity at baseline did not differ in baseline GFR from the group with high activity. A simple physical activity scale, as used in the present study, could guide health care personnel in prescribing specific exercise interventions for these patients.

Overall, at the group level, the individuals with CKD in our study were very stable in all measured parameters: i.e., cardiovascular, muscular and haematological. Cardiac function and vascular stiffness showed no significant change over time in the CKD group, while the controls significantly decreased their cardiac diastolic and systolic function and their peak HR. The changes in the control group can be considered being the result of normal aging. Data from our group show that in the same cohort, daytime systolic blood pressure did not increase over 5 years in the CKD group, while a small increase was seen in the controls [23]. Aerobic ExCap is dependent on the function of the cardiovascular system and skeletal musculature. Given the lack of progression in outcomes representing the function of these systems, it is not surprising that aerobic ExCap did not decrease significantly in the CKD group.

The participants with mild-to-moderate CKD in our study were recruited from a dedicated nephrology clinic and showed well-controlled blood pressure over time [23]. The CKD group was closely followed and underwent vigorous treatment of risk factors for cardiovascular disease. These factors may explain the fact that this group could maintain their cardiovascular and muscular function and thereby their aerobic ExCap, however these findings should be confirmed in a randomized study. The mean decline in GFR over time was comparable to or a little lower than values reported in other studies [12, 35, 36]. Interestingly, Jones et al. reported that GFR decline slowed significantly following referral to a nephrology clinic, indicating that interventions to slow progression of CKD are important and beneficial. The decline of GFR in the control group was not more pronounced than expected in healthy individuals [37].

Strengths of this study are the prospective design with a control group and that we analysed changes in aerobic ExCap, physical activity as well as cardiovascular function at the same time. Given the scarce data available on longitudinal changes in ExCap in CKD, our study adds valuable information.

One limitation to our study is the relatively small study population at baseline and that some individuals were lost to follow-up. The choice of statistical method was intended to compensate for the loss of subjects during the follow-up period. Aerobic ExCap was measured as peak workload and not VO_2_peak, the golden standard method to assess aerobic ExCap and if the test is truly maximal. During cycle ergometry, there is a linear relationship between exercise load and oxygen uptake [38] and the tests were performed according to the same protocol at both time-points with similar high RPEs. Therefore, we believe our results are valid measurements of the true changes in aerobic ExCap and peak HR over time.

## Conclusions

On a group level, aerobic ExCap and peak HR were maintained over 5 years in mild-to-moderate CKD, despite a slight decline in renal function. In line with the maintained ExCap, cardiovascular and muscular function were also preserved. Early referral to a specialized nephrology unit may have contributed to the preserved aerobic, cardiovascular and muscular function.

Using a simple physical activity scale, patients with low physical activity can be recognized as a risk group for more rapid decline in exercise capacity and possibly further deterioration in every day physical function. This can be targeted by specific exercise interventions in these patients. Further longitudinal studies with long-term objective monitoring of physical activity could provide more clarity on the importance of physical activity in maintaining aerobic ExCap in CKD.

## Data Availability

The datasets used and/or analysed during the current study are available from the corresponding author on reasonable request.

## Abbreviations

CI: Confidence interval
CKD: Chronic kidney disease
CKD–EPI: Chronic Kidney Disease–Epidemiology Collaboration
E/é: Mitral flow velocity E divided by tissue LV velocity é
EF: Ejection fraction
eGFR: Estimated GFR
Ep: Pressure strain elastic modulus in the carotid artery
ExCap: Exercise capacity
GEE: Generalized estimating equations
GFR: Glomerular filtration rate
Hs-CRP: High-sensitivity C-reactive protein
HR: Heart rate
LBM: Lean body mass
LV: Left ventricular
OR: Odds ratio
RPE: Rating of perceived exertion
TAPSE: Tricuspid annular plane systolic excursion
VO_2_peak: Peak oxygen uptake
